# *Blastocystis* sp. Prevalence and Subtypes Distribution amongst Syrian Refugee Communities Living in North Lebanon

**DOI:** 10.3390/microorganisms9010184

**Published:** 2021-01-16

**Authors:** Salma Khaled, Nausicaa Gantois, Aisha Ayoubi, Gaël Even, Manasi Sawant, Jinane El Houmayraa, Mathieu Nabot, Sadia Benamrouz-Vanneste, Magali Chabé, Gabriela Certad, Dima El Safadi, Fouad Dabboussi, Monzer Hamze, Eric Viscogliosi

**Affiliations:** 1 University Lille, CNRS, Inserm, CHU Lille, Institut Pasteur de Lille, U1019—UMR 9017—CIIL—Centre d’Infection et d’Immunité de Lille, F-59000 Lille, France; salmakhaled94@outlook.com (S.K.); nausicaa.gantois@pasteur-lille.fr (N.G.); manasi.sawant@pasteur-lille.fr (M.S.); sadia.benamrouz@univ-catholille.fr (S.B.-V.); magali.chabe@univ-lille.fr (M.C.); gabriela.certad@pasteur-lille.fr (G.C.); 2 Laboratoire Microbiologie Santé Environnement (LMSE), Ecole Doctorale des Sciences et Technologie, Faculté de Santé Publique, Université Libanaise, Tripoli, Lebanon; aishaayoubi91@gmail.com (A.A.); dima.elsafadi@hotmail.com (D.E.S.); fdaboussi@hotmail.com (F.D.); mhamze@monzerhamze.com (M.H.); 3 Gènes Diffusion, F-59501 Douai, France; g.even@genesdiffusion.com; 4 PEGASE-Biosciences (Plateforme d’Expertises Génomiques Appliquées aux Sciences Expérimentales), Institut Pasteur de Lille, F-59000 Lille, France; 5 Concern Worldwide, HDYS Building (Opposite Abdel Karim Rifai Petrol Station), Halba, Akkar, Lebanon; jinane.elhoumayraa@concern.net; 6 Solidarités International, Ras Al Naba’a, Chaar street, Chaar building, Beirut, Lebanon; cdm@solidarites-liban.org; 7 Laboratoire Ecologie et Biodiversité, Faculté de Gestion Economie et Sciences, Institut Catholique de Lille, F-59000 Lille, France; 8 Délégation à la Recherche Clinique et à l’Innovation, Groupement des Hôpitaux de l’Institut Catholique de Lille, F-59462 Lomme, France

**Keywords:** *Blastocystis* sp., intestinal parasites, Syrian refugees, North Lebanon, internal tented settlements, molecular epidemiology, real-time PCR, SSU rDNA subtyping, transmission, risk factors

## Abstract

Molecular data concerning the prevalence and subtype (ST) distribution of the intestinal parasite *Blastocystis* sp. remain scarce in the Middle East. Accordingly, we performed the first molecular epidemiological survey ever conducted in the Syrian population. A total of 306 stool samples were collected from Syrian refugees living in 26 informal tented settlements (ITS) subjected or not to water, sanitation, and hygiene (WASH) interventions in North Lebanon, then screened for the presence of *Blastocystis* sp. by real-time polymerase chain reaction followed by subtyping. The overall prevalence of the parasite was shown to reach 63.7%. *Blastocystis* sp. colonization was not significantly associated with gender, age, symptomatic status, abdominal pain or diarrhea. In contrast, WASH intervention status of ITS was identified as a risk factor for infection. Among a total of 164 subtyped isolates, ST3 was predominant, followed by ST1, ST2, and ST10. No particular ST was reported to be associated with age, gender, symptomatic status, digestive disorders, or WASH intervention status of ITS. Intra-ST diversity of ST1 to ST3 was low suggesting large-scale anthroponotic transmission. Moreover, comparative analysis of ST1 to ST3 genotypes revealed that the circulation of the parasite between Syrian refugees and the host population was likely limited.

## 1. Introduction

*Blastocystis* sp. is currently the most frequent intestinal protozoa found in the human gut [1,2,3,4]. Indeed, by extrapolating published prevalence data from many countries, *Blastocystis* sp. is estimated to colonize between 1 and 2 billion people on a global scale [5]. This high prevalence of the parasite all over the world is strongly related to its main mode of transmission, which is the fecal-oral route mainly through the consumption of contaminated water [6,7]. The transmission of *Blastocystis* sp. is thus naturally favored by the precarious sanitary and hygienic conditions encountered in certain populations with low level of sanitation, particularly in African, Asian, or American developing countries [8,9,10,11,12,13]. On the other hand, the zoonotic potential of *Blastocystis* sp. has been confirmed in recent epidemiological surveys and several animal groups colonized by the parasite such as birds and pigs may represent additional reservoirs of infection for human beings [14,15,16], thus potentially contributing to a prevalence increase.

Consequently, the ubiquity and high prevalence of the parasite has a major impact on public health even though a large number of individuals colonized by the parasite do not present digestive symptoms (i.e., asymptomatic patients) [7,17,18,19]. However, asymptomatic carriage and high prevalence in healthy individuals does not preclude pathogenicity. Indeed, numerous clinical cases of blastocystosis reported patients presenting nonspecific gastrointestinal disorders including diarrhea and abdominal pain, without any other causal organisms [20,21]. It is therefore clear that, under certain conditions related for instance to the microbiota composition and immunocompetent status of the host and/or to the variable virulence of the parasite isolates, colonization by *Blastocystis* sp. becomes an infection. In this regard, virulence factors and mechanisms associated with the pathogenicity of the parasite have been identified mainly through the accumulating data of in vitro and comparative genomics studies [22,23].

Even if it remains challenging to differentiate one *Blastocystis* sp. isolate from another based on morphological criteria [1,2,7], this genus is highly genetically divergent since 17 different subtypes (STs) (arguably species) have been validated so far in mammalians and birds through the comparison of small subunit (SSU) rDNA gene sequences [24,25,26]. This genetic diversity also extends to *Blastocystis* sp. isolates identified in amphibians, reptiles, fish and insects, in which potential additional STs were described [14,27,28,29,30]. By synthesizing all available epidemiological data, *Blastocystis* sp. ST1 to ST4 represent the most frequent STs identified in the human population worldwide and account for around 95% of all human carriage [9,18,31,32,33], consistent with a large-scale anthroponotic transmission of the parasite. The eight other STs found in the human population with a lower frequency (ST5–ST10, ST12 and ST14) [9,14,32,34] were supposed to be of zoonotic origin. This was strongly evidenced through the unusually high prevalence of ST8 in zoo monkey keepers [34], ST5 in piggery workers [15] and ST6 in poultry slaughterhouse staff [16], likely correlated with repeated exposure to animals.

Despite an increasing number of epidemiological surveys conducted in the last decade to clarify the molecular distribution and genetic diversity of *Blastocystis* sp. isolates around the globe, several geographical regions remain yet poorly explored such as the Middle East and Arabian Gulf countries. Indeed, data currently available mainly focuses on Lebanon [4,16,35,36], Iran [37,38], and Saudi Arabia [39]. To our knowledge, no molecular epidemiological study has ever been performed in the Syrian population, in part because of the humanitarian crisis that has heavily impacted this country since 2011. This crisis has furthermore displaced a huge number of refugees into Lebanon increasing the total host population by 30% [40]. Most Syrian refugees are at risk of contracting parasitic infections as they face extremely high rates of poverty without proper access to infrastructure services and adequate sewage systems. This may obviously create the perfect environment for wide dissemination and outbreaks of parasitic infections among Syrians and Lebanese population as well.

Therefore, our first aim was to fill a geographical gap in the knowledge of *Blastocystis* sp. prevalence and diversity by conducting a large-scale epidemiological survey in Syrian refugees living in informal tented settlements (ITS) in North Lebanon and to assess the burden of the parasite in this at-risk population. In addition, since molecular data regarding *Blastocystis* sp. epidemiology were already available from previous studies conducted in the Lebanese population living in the North Lebanon region, our second aim was thus to compare the STs and genotypes identified in the Syrian and local populations to investigate the circulation/transmission of the parasite between both communities.

## 2. Materials and Methods

### 2.1. Ethics Approval

Ethical approval for this project was obtained from the local competent Ethics Committee of AZM Center for Research in Biotechnology and its Applications, Tripoli, Lebanon (Reference number CE-EDST-7-2018; Date of approval: 12/14/2018). This study was conducted in accordance with the Code of Ethics of the World Medical Association (Declaration of Helsinki III) and with the International Ethical Guidelines for Biological Research Involving Human Subjects. After a clear explanation of the research objectives prior to enrolment, written informed consent was obtained from each adult or from the parents or legal guardians of each child included in this study.

### 2.2. Questionnaire Survey

A standardized questionnaire was designed to collect information about each participating Syrian refugee including gender, age and city of origin as well as clinical data regarding the presence of any digestive symptoms (diarrhea, abdominal pain, vomiting, bloating and constipation). A participant was considered symptomatic if at least one of the five selected digestive disorders described above was present. All data collected from each subject remained confidential and were fully anonymized through the encryption of the identity of individuals.

### 2.3. Sites Sampling and Collection of Samples

**Figure 1:**
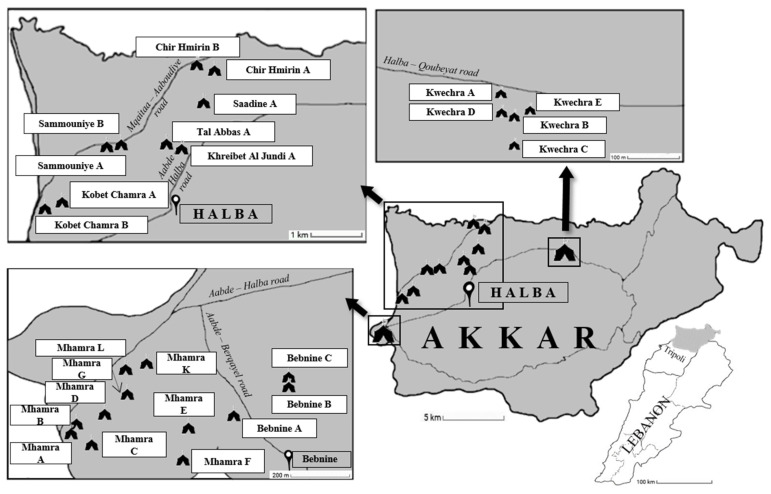
Location of the 26 ITS in the governorate of Akkar in the North Lebanon region screened for the presence of *Blastocystis* sp.

This large-scale study was conducted in 26 Informal Tented Settlements (ITS) inhabited by Syrian refugees and located in the vicinity of the city of Halba (latitude 34°33′2″ N, longitude 36°4′41″ E), the capital of the Akkar governorate in North Lebanon region (Figure 1). The nomenclature of the ITS was also encrypted in the present report.

Halba is situated about ten kilometers from the Syrian border. The refugees participating in this survey originate from several major Syrian cities such as Aleppo, Racca, Homs, Hama, Qamishli and Deir ez-Zor. The distribution of the refugees in the ITS is mainly organized by region of origin and a large proportion of them are housed in these ITS since the beginning of the Syrian crisis in 2011. As shown in Table 1, 184 stool samples were collected in 16 ITS so-called ITS with Water, Sanitation and Hygiene (WASH) intervention (WI), in which drinking water supply, rehabilitation/construction of toilets, regular waste removal and hygiene promotion awareness were provided by the two non-governmental organizations (NGOs) Concern Worldwide and Solidarités International. The remaining 122 stool samples were collected in 10 other ITS so called ITS without WI where no facilities were yet provided by NGOs at the time of sampling regarding sanitation and hygiene conditions. For drinking, washing, and domestic activities, refugees in ITS without WI could use wells close to the camps. In the absence of wells, NGOs provide only drinking water through safe water trucking.

With the valuable assistance of NGOs staff, the fecal sample collection was conducted between February and December 2019. Briefly, 1 to 58 stool samples were collected in each of the 26 ITS (Table 1), for an overall total of 306 samples. The highly variable number of collected samples between ITS was correlated to the size of the settlements and the number of volunteers for this study in each sampling site. For each subject, one fresh stool sample was collected in a sterile container then immediately transported to the Laboratory Microbiologie Santé et Environnement, Doctoral School of Sciences and Technologie in Tripoli for DNA extraction.

### 2.4. DNA Extraction and Molecular Subtyping of Blastocystis sp. Isolates

Total genomic DNA was extracted from approximately 250 mg of stool samples using the QIAamp DNA Stool Mini Kit (Qiagen GmbH, Hilden, Germany) according to the manufacturer’s recommended procedures then eluted in 200 μL of elution buffer (Qiagen GmbH, Hilden, Germany). All DNA samples were stored at −20 °C then transported to the Institut Pasteur of Lille (Lille, France). To detect and subtype *Blastocystis* sp., 2 µL of extracted DNA from each sample was subjected to highly sensitive real-time PCR (qPCR) assay using the *Blastocystis*-specific primers BL18SPPF1 (5′-AGTAGTCATACGCTCGTCTCAAA-3′) and BL18SR2PP (5′-TCTTCGTTACCCGTTACTGC-3′) targeting the small subunit (SSU) rDNA gene as previously described [41]. The corresponding amplified gene domain of about 300 bp has been shown to contain sufficient sequence information for accurate subtyping of *Blastocystis* sp. isolates. Positive (DNA from *Blastocystis* sp. ST7 axenic culture) and negative (DNA replaced by water) qPCR controls were included with each batch of samples analyzed. The qPCR product from each positive sample was purified and directly sequenced on both strands (Genoscreen, Lille, France). For a proportion of samples, sequence chromatograms analysis revealed the presence of double traces, suggesting mixed infections by at least two different *Blastocystis* STs that were not determined. The SSU rDNA sequences obtained in this study from samples presenting single infection were deposited in GenBank under accession numbers MW168447 to MW168610. Obtained sequences were compared with all *Blastocystis* sp. homologous sequences of known STs available from the National Centre for Biotechnology Information (NCBI) using the nucleotide Basic Local Alignment Search Tool (BLAST) program. STs were identified by determining the exact match or closest similarity against all known *Blastocystis* sp. STs [14,26]. Moreover, the sequences of *Blastocystis* sp. isolates belonging to the same ST (ST1, ST2 or ST3) were aligned with each other using the BioEdit v7.0.1 package (Date of release 06/10/2019; http://www.mbio.ncsu.edu/BioEdit/bioedit.html) to determine intra-ST diversity and identify so-called genotypes referring to genetically distinct strains within the same ST as described in recent surveys [9,16]. Subsequently, ST1, ST2 and ST3 sequences from isolates previously identified in the local population of North Lebanon [4,16,35,36] were extracted from databases and compared to genotypes reported herein from the cohort of Syrian refugees.

### 2.5. Statistical Analysis

For the statistical analysis, Fisher’s exact test was used to test the relationship between different categorical variables. Multilevel logistic mixed regression models were also used to calculate odds ratios (OR) and 95% confidence interval (CI) and to measure the association of the explanatory variables considering *Blastocystis* sp. colonization, STs and genotypes as the main outcomes. The general significance level was set at a *p*-value below 0.05. All analyses were performed using packages stats and odds ratio from the R statistical computing program v. 3.6.1 (Date of release 07/05/2019) (R Development Core Team; http://www.R—project.org).

## 3. Results

### 3.1. Analysis of the Cohort of Syrian Refugees and Prevalence of Blastocystis sp.

Stool samples were collected from a total of 306 Syrian refugees housed in 26 ITS located in North Lebanon. The sex ratio (F/M) was 1.47 and the age of the participants was between one and 85 years (mean age of 20.4 ± 16.7 years). Epidemiological records revealed that 131/306 refugees (42.8%) suffered from digestive symptoms. Among this cohort, 74 individuals (56.5%) presented abdominal pain, 47 (35.9%) diarrhea, 36 (27.5%) constipation, 32 (24.4%) bloating and seven (5.3%) vomiting. Moreover, 84 of the 131 symptomatic individuals reported only one of these digestive symptoms, while 31 and 16 exhibited two or three of these selected intestinal disorders, respectively. The remaining 175 participants were considered asymptomatic at the time of the survey. The number of asymptomatic individuals was statistically significantly higher than the number of symptomatic individuals in the age groups 0–14 years (101 asymptomatic versus 56 symptomatic; OR: 0.547, CI: 0.345–0.863, *p* = 0.001) but not in the 15–29 years age group (35 versus 29; OR: 1.137, CI: 0.65–1.978, *p* = 0.6). In contrast, the prevalence of symptomatic participants was reported to be significantly higher in the group aged over 30 years (46 versus 39; OR: 1.887, CI: 1.14–3.14, *p* = 0.01)

### 3.2. Screening for Blastocystis sp. Using qPCR

Of the 306 stool samples tested for the presence of *Blastocystis* sp. by qPCR, 195 of them were positive for the parasite leading to an overall prevalence of 63.7% (Table 1). Analysis of our data based on qPCR detection revealed that neither sex (62.9% of males versus 64.3% of infected females; OR: 1.062, CI: 0.659–1.704, *p* = 0.80) nor age (Fisher exact test, *p* = 0.1) of the participants affected prevalence significantly. However, subgroup analysis showed the highest prevalence of *Blastocystis* sp. in the group of refugees aged over 30 years (60/85, 70.6%), followed by the group aged 0–14 years (101/157, 64.3%) and subjects aged 15–29 years (34/64, 53.1%). The average prevalence of the parasite in refugees housed in ITS without WI (86/122, 70.5%) was significantly higher than that observed among refugees living in ITS with WI (109/184, 59.2%) (OR: 1.644, CI: 1.014–2.695, *p* = 0.04). Regarding *Blastocystis* sp. infection and digestive symptoms, the prevalence of the parasite was not significantly higher in symptomatic refugees (84/131, 64.1%) than in asymptomatic carriers (111/175, 63.4%) (OR: 1.03, CI: 0.644–1.655, *p* = 0.9). Within the group of participants positive for *Blastocystis* sp., abdominal pain was reported in 27.2% (53/195) of the subjects, followed by diarrhea (17.4%, 34/195), constipation (9.2%, 18/195), bloating (8.7%, 17/195) and vomiting (1.5%, 3/195). Even if abdominal pain and diarrhea were more frequent in this cohort compared to non-carriers (27.2% versus 18.9% and 17.4% versus 11.7%, respectively), no significant difference was identified in the frequency of these symptoms between these two groups of participants (OR: 1.6, CI: 0.915–2.876, *p* = 0.11 for abdominal pain and OR: 1.592, CI: 0.818–3.265, *p* = 0.18 for diarrhea).

### 3.3. Distribution of Blastocystis sp. STs and Genotypes

The qPCR products of the 195 samples positive for *Blastocystis* sp. in the present study were all sequenced on both strands. For 31 of these samples (15.9%), sequence chromatograms with multiple signals were obtained, suggesting mixed infections by different STs. The remaining 164 isolates corresponded to single infections by one ST, exhibiting 100% sequence identity with the reference sequences of known STs as for instance isolate 26 sequence for ST1 (GenBank accession number MT645808), isolate 515 sequence for ST2 (MN836830), isolate 31 sequence for ST3 (MT645813) and isolate HC104 for ST10 (MF573945). The most frequent ST identified in the entire cohort of Syrian refugees was ST3 (54.3%; 89/164), followed by ST1 (26.2%; 43/164), ST2 (18.9%; 31/164) and ST10 (0.6%; 1/164). This ST distribution was not significantly associated with gender (Fisher exact test, *p* = 0.29) or age (Fisher exact test, *p* = 0.19) of the refugees. No significant difference in the distribution of STs was reported between ITS with or without WI because ST1 (OR: 1.651, CI: 0.836–3.186; *p* = 0.15), ST2 (OR: 0.789, CI: 0.351–1.716, *p* = 0.56) and ST3 (OR: 0.863, CI: 0.486–1.528, *p* = 0.61) were not more frequently found in either of these two types of ITS. No significant association was detected between ST1 (OR: 0.505, CI: 0.237–1.028, *p* = 0.07), ST2 (OR: 1.082, CI: 0.494–2.342, *p* = 0.84) or ST3 (OR: 1.705, CI: 0.962–3.041, *p* = 0.07) and the symptomatic status of the refugees. Abdominal pain and diarrhea were also not shown to be significantly associated with ST (Fisher exact tests, *p* = 0.45 and *p* = 0.76, respectively).

**Figure 2:**
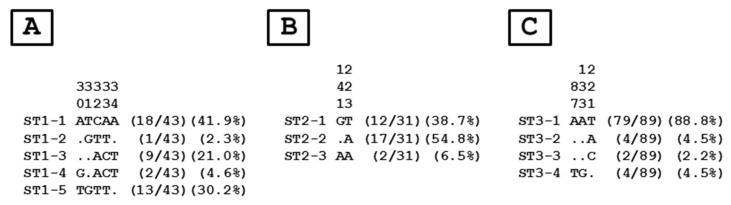
Alignment of partial SSU rDNA gene sequences from *Blastocystis* sp. ST1 (**A**), ST2 (**B**), and ST3 (**C**) isolates colonizing Syrian refugees. Positions of variable nucleotides in comparison to reference sequences (genotypes ST1-1, ST2-1 and ST3-1) are indicated above the alignment (vertical numbering). Genotypes identified within each ST are indicated on the left of the alignments. Nucleotides identical to those of the reference sequences are represented by dashes. On the right of each alignment are reported the total number and percentage of isolates identified in Syrian refugees for each genotype.

To evaluate intra-ST diversity and identify genotypes, the partial SSU rDNA gene sequences obtained in the present survey and belonging to ST1, ST2 or ST3 were aligned with each other. As shown in Figure 2, the comparison of the 43 ST1, 31 ST2, and 89 ST3 sequences revealed five, two, and three variable positions, respectively, i.e., positions showing at least one nucleotide difference within at least one of the compared sequences. This comparison allowed the identification of five ST1 (ST1-1 to ST1-5), three ST2 (ST2-1 to ST2-3), and four ST3 (ST3-1 to ST3-4) genotypes among the Syrian population.

The ratio between the number of isolates and genotypes belonging to ST1, ST2 and ST3 was 8.6 (43/5), 10.3 (31/3) and 22.3 (89/4), respectively. The major ST1 (ST1-1, ST1-3 and ST1-5), ST2 (ST2-1 and ST2-3), and ST3 (ST3-1) genotypes included all together more than 90% of the total number of isolates characterized in this study. The remaining 2 ST1, 1 ST2 and 3 ST3 genotypes were only represented by 1–4 isolates. The distribution of genotypes was not significantly different according to age (Fisher exact test, *p* = 0.30), WI status of ITS (Fisher exact test, *p* = 0.39), symptomatic status of the refugees (Fisher exact test, *p* = 0.21) or digestive disorders exhibited by Syrian participants such as abdominal pain (Fisher exact test, *p* = 0.58) and diarrhea (Fisher exact test, *p* = 0.76). In contrast, distribution of genotypes was reported to be slightly significantly associated with sex of the refugees (Fisher exact test, *p* = 0.05). Accordingly, genotype ST3-1 which largely predominated in refugees was less frequently identified in males than in females (OR: 0.513, CI: 0.267–0.971, *p* = 0.04). Moreover, although the number of isolates belonging to genotype 1–5 was limited, this genotype was more frequent in males than in females (OR: 3.958, CI: 1.227–15.172, *p* = 0.03)

To clarify the circulation of *Blastocystis* sp. between refugees and the host population, the 277 sequences of isolates previously identified in Lebanese individuals living in the region of North Lebanon and belonging to ST1 (68 isolates), ST2 (74 isolates), and ST3 (135) (Table 2) were extracted from databases and aligned with those obtained in the present study.

Briefly, among the 68 Lebanese ST1 isolates, 22 of them (32.4%) showed identical sequences with Syrian ST1-1 (10), ST1-2 (2), ST1-3 (5) or ST1-5 (5) genotypes. Syrian genotype ST1-4 was not found in the Lebanese population. Regarding the 74 Lebanese ST2 isolates, 19 of them (25.7%) exhibited 100% sequence identity with Syrian ST2-1 (12), ST2-2 (6) or ST2-3 (1) genotype. Strikingly, only 3.7% of the Lebanese ST3 isolates (5/135) belonged to ST3-2 (1) and ST3-3 (4) Syrian genotypes. The predominant Syrian genotype ST3-1 together with ST3-4 was not identified in the Lebanese population.

## 4. Discussion

To our knowledge, this study is the first to explore the prevalence and distribution of *Blastocystis* sp. STs and genotypes in the Syrian population. This allowed us to broaden our knowledge about the epidemiology of *Blastocystis* sp. in a region of the world still poorly investigated, such as the Middle East, wherein some populations living in this geographical area may be considered at risk of infection due to precarious sanitary conditions and deficient drinking water supply. Because of the Syrian crisis, conducting field studies in Syria remains almost impossible which may partly explain the crucial lack of current molecular epidemiological data and burden assessment regarding intestinal parasitic protozoa. Consequently, this large-scale survey was conducted in the ITS housing Syrian refugees in the North Lebanon region.

Through the screening of stool samples collected from 306 Syrian refugees of which about 43% suffering from digestive symptoms, qPCR assay revealed an overall prevalence of *Blastocystis* sp. reaching 63.7%, highlighting a wide circulation of the parasite in this community. Such high frequency corresponded well with prevalence data obtained previously using molecular tools in Middle Eastern countries such as Lebanon (55 to 63% depending of the cohort) [4,16,36], but was much more substantial than those observed in Saudi Arabia (10.5%) [39] and Iran (6.5%) [42]. Prevalence of *Blastocystis* sp. in Syrian refugees was also of the same order of magnitude as those reported in Qatar (71.1%) [43] and the United Arabian Emirates (UAE) (44.4%) [44]. However, these two latter studies did not reflect the situation of the parasite in the local populations since the small size cohorts analyzed corresponded to migrant workers newly arriving in Qatar and UAE and originating from African, East Asian and West Asian countries.

Among the overall Syrian cohort, gender was not identified as a potential risk factor associated with *Blastocystis* sp. colonization, considering that both males and females have highly similar values of prevalence. This statement was in agreement with previous epidemiological surveys conducted in Middle Eastern countries [4,43,44]. In the same way, no significant difference in prevalence of the parasite between the age classes in our data (0–14 years, 15–29 years, and over 30 years) was reported. However, the prevalence of *Blastocystis* sp. was higher above the age of 30 years which could be explained by frequent reinfections or stable long term colonization of these adults by the parasite. Not surprisingly, WI status of ITS represented a risk factor for *Blastocystis* sp. infection, with prevalence of the parasite significantly higher in ITS without WI. This difference could easily be explained by the poor hygiene conditions and low level of water safety in ITS without WI, thus facilitating the transmission of the parasite. Indeed, intestinal parasites are usually considered as vectors of poverty related diseases as highlighted for instance by the high prevalence of *Blastocystis* sp. revealed in low-income African [9], Asian [11], and American countries [13]. Moreover, the prevalence of *Blastocystis* sp. was for instance reported to be significantly higher in schoolchildren of low socioeconomic status than in schoolchildren of high socioeconomic status in Lebanon [4]. In parallel, accumulating epidemiological data do not support a clear association between *Blastocystis* sp. infection and the presence of digestive symptoms [3,7,17] and this is also the case among the overall refugee population of the present study. Moreover, no digestive symptoms were significantly identified more frequently within the cohort of *Blastocystis* sp. carriers. Nevertheless, abdominal pain was recorded as the most common digestive disorder in Syrian refugees colonized by the parasite as described in earlier studies [4,7,45].

Among the stool samples positive for *Blastocystis* sp. in the present survey, 15.9% of them (31 in 195) represented mixed infections consisting of at least two different STs according to the resulting sequence chromatograms. This relevant percentage revealed that mixed infections were common in the Syrian refugees as it was previously demonstrated in other cohorts [9,46], in link with a probable exposure of individuals to different sources of infection. In the ST analysis of the remaining 164 positive isolates corresponding to single infections, ST3 was the most commonly detected ST, followed by ST1 and ST2. This predominance of ST3, which is highly common in the human population worldwide [17,18,26], is also emphasized in the compiled studies from Lebanon (Table 2) along with the neighboring countries including Iran [37,47,48] and Saudi Arabia [39]. The only difference in ST distribution observed between Middle Eastern cohorts is that the ST1 was the second most common variant after ST3 within the Syrian refugees and in Saudi Arabia while it stands at the third position among the overall subtyped Lebanese isolates, but still with a high frequency. Since ST1 to ST3 are predominant in the human population [26] and less frequently identified in animal groups [14], this strongly suggested that Syrian refugee infections by these STs were mostly correlated with large-scale inter-human transmission. Interestingly, ST4 with the focus being in Europe [41,45,49] was completely absent among individuals participating to this survey. In Middle Eastern countries, only one ST4 isolate has been previously identified in Lebanon [35] and no more than two dozen on a total of around 700 subtyped isolates in Iran [50,51,52]. In addition, it was pointed out that ST4 seems rare in regions with mainly Muslim population [26], situation that is highly probable in the current study, hypothesizing a potential link with eating habits or certain animal contacts of this population. Why ST4 is absent in certain areas of the world remains unknown although the hypothesis of a recent emergence of this ST in Europe probably associated with a zoonotic origin has been proposed [17]. Interestingly, the last isolate subtyped in Syrian refugees belonged to ST10. Until very recently, this ST had never been documented in humans worldwide, whereas it represents the most widely distributed ST in bovid [14,36]. In a large epidemiological study conducted in Africa [9], ST10 isolates were reported in two Senegalese individuals. As for the zoonotic ST10 isolate identified in the present study, the transmission of this ST may be due to direct contact with livestock or consumption of water contaminated by ST10 parasite cysts.

Furthermore, ST distribution of *Blastocystis* sp. was similar between ITS with or without WI and no significant association was demonstrated between ST and gender or age of the participants. Interestingly, no particular ST was consistently linked to the symptomatic status of refugees or digestive disorders such as abdominal pain or diarrhea. Numerous molecular studies have investigated the possibility of a link between *Blastocystis* sp. STs and symptoms and the corresponding results were contradictory [7,17], probably for two main reasons. The first reason is that it is almost impossible to exclude any other identifiable cause (other intestinal parasites, bacteria or virus) of the same digestive symptoms as those described for blastocystosis for each individual within a population. The second reason is that the clinical outcome of *Blastocystis* sp. infection by the same isolate from one individual to another may largely depend on the immunity and composition of the intestinal microbiota of the host [53] and on the genotype/virulence [22] of the parasite. Consequently, individual clinical case studies will remain the main source of information focusing on the potential link between ST and symptoms [20].

The next step of our survey was to investigate the intra-ST diversity of the three major STs (ST1 to ST3) reported in the Syrian refugee cohort. This diversity was determined through the identification of genotypes based on nucleotide differences at the SSU rDNA locus among isolates and the evaluation of the ratio between the number of isolates and the number of genotypes as previously described [9]. ST1 was delineated to 5 different genotypes (ST-1 to ST1-5) with an average of 8.6 isolates per genotype. In case of ST2 and ST3, the number of genotypes was respectively three and four, with an average ratio of 10.3 for ST2 and 22.3 for ST3. Considering only the number of genotypes, ST2 would appear to be the ST with the lower intra-ST diversity. However, this statement was invalidated by the analysis of the average isolates/genotypes ratios highlighting that ST3 had the lower intra-ST diversity in the present study followed by ST2 and ST1. Strikingly, the same approach targeting this domain of the 18S rDNA locus showed a similar pattern of intra-ST diversity for ST1 to ST3 among Senegalese isolates [9]. In addition, the lower intra-ST diversity of ST3 was also confirmed through the comparative analysis of draft *Blastocystis* sp. genomes from various STs [54]. None of the Syrian genotypes belonging to ST1, ST2 or ST3 was more frequently found according to age or WI status of ITS. Similarly, no genotype was significantly associated with the symptomatic status of the refugees or with the presence of abdominal pain or diarrhea.

In the Syrian cohort, the number of genotypes characterized was undoubtedly very low compared to the above-mentioned recent study conducted in Senegal [9]. Indeed, a total of 43 different genotypes were reported within the Senegalese cohort with a significant proportion of these genotypes represented by a limited number of isolates. These observations suggested the existence of multiple potential environmental sources of infection by the parasite in this rural region of Senegal in addition to large anthroponotic transmission. Within the Syrian cohort, it was therefore evident that human-to-human transmission was largely predominant, thus explaining the low number of genotypes found in this population. This could be explained on the one hand by the relatively low number of children enrolled in Lebanese schools (around 25%), who additionally attend school at different hours than Lebanese schoolchildren, thus limiting contacts between these two populations. On the other hand, only 15% of adult refugees have a professional activity outside the camp that is mostly related to agriculture in the vicinity of the settlements. Both statements could thus favor human-to-human transmission of the parasite among refugees within the ITS.

This assumption was also supported by the thorough comparative analysis of the *Blastocystis* sp. genotypes identified in Syrian refugees and those circulating in the local Lebanese population of North Lebanon. Strikingly, only 16.6% of the 277 Lebanese ST1 to ST3 isolates (Table 2) could be classified among the 12 Syrian genotypes. Moreover, Syrian genotypes ST1-4, ST3-4, and especially ST3-1 were not identified in the Lebanese population. On this basis, we can hypothesize that the circulation/transmission of the parasite between local and refugee populations was quite restricted because of limited contact. Accordingly, genotype ST3-1 which is largely predominant in the Syrian cohort with around 90% of ST3 isolates was significantly more frequently identified in females. A suggested explanation may be that women are more likely to stay longer in the ITS during the day even though, like men, they also leave the settlements for work activities. Consequently, inter-human transmission undoubtedly perpetuates the over-representation of this genotype, especially in this group of refugees and promiscuity in some of the ITS obviously facilitates such a transmission. In contrast, genotype ST1-5 which represents only about 30% of the Syrian ST1 isolates was overrepresented in males. Since this genotype has also been reported in the Lebanese population, it can therefore be proposed that Syrian males harboring ST1-5 could have been contaminated through an external source as a result of their participation in outdoor activities. Another underlying hypothesis that could be tested in future epidemiological studies would be the circulation of this genotype in the different countries of the Middle East and the Arabian Gulf.

## 5. Conclusions

The present survey is the first to provide epidemiological data on the prevalence and distribution of *Blastocystis* sp. STs and genotypes in the Syrian population, giving a more comprehensive view of the parasite burden in Middle Eastern countries. The prevalence observed in our cohort of Syrian refugees living in ITS in North Lebanon is significant since it exceeds 60%, highlighting the active circulation of the parasite in this population. Moreover, by comparing the prevalence of the parasite in ITS with or without WI, it is clearer that poorer access to water, sanitation and hygiene promotion awareness favor the transmission of *Blastocystis* sp. in such an environment. In parallel, the comparative analysis of the STs and genotypes identified in the Syrian refugees and local North Lebanese population strongly suggests that *Blastocystis* sp. is mainly transmitted through the inter-human route in the Syrian cohort and that the circulation of the parasite between the refugee and host communities remains limited. Screening programs of intestinal protozoa together with the implementation of prevention and health education measures, sanitary facilities and water quality monitoring are thus urgently needed in the ITS to improve the health status of the refugees and prevent the spread of the parasites among refugees and to the host population.

## Figures and Tables

**Table 1:** Prevalence and ST distribution of *Blastocystis* sp. in the ITS housed by Syrian refugees screened in the present study.

ITS ^a^	Samples (*n*)	Positive Samples (*n*)	Prevalence (%)	*Blastocystis* sp. STs				
				ST1	ST2	ST3	ST10	MI ^b^
**With WI ^c^**								
Kwechra A	1	0	0	0	0	0	0	0
Kwechra B	6	6	100	2	1	2	0	1
Kwechra C	7	4	57.1	0	0	2	0	2
Kwechra D	13	12	92.3	4	2	4	0	2
Kwechra E	7	5	71.4	1	0	4	0	0
Mhamra A	16	8	50	2	0	6	0	0
Mhamra B	21	15	71.4	3	0	11	0	1
Mhamra C	39	21	53.8	2	8	5	0	6
Mhamra D	14	7	50	2	3	1	0	1
Mhamra E	17	10	58.8	1	0	7	0	2
Chir Hmirin A	3	3	100	2	0	1	0	0
Chir Hmirin B	2	2	100	0	0	2	0	0
Khraybet Al Jundi A	16	9	56.3	1	3	3	0	2
Sammouniye A	3	2	66.7	0	0	1	0	1
Saadine A	17	5	29.4	0	2	3	0	0
Tal Abbas A	2	0	0	0	0	0	0	0
Total	184	109	59.2	20	19	52	0	18
**Without WI**								
Bebnine A	8	4	50	2	0	1	0	1
Bebnine B	10	5	50	2	1	2	0	0
Bebnine C	1	0	0	0	0	0	0	0
Mhamra F	4	4	100	1	2	0	0	1
Mhamra G	6	4	66.7	0	1	2	1	0
Mhamra K	2	2	100	0	0	2	0	0
Mhamra L	6	5	83.3	2	1	2	0	0
Kobet Chamra A	16	11	68.8	4	1	6	0	0
Kobet Chamra B	58	43	74.1	9	3	20	0	11
Sammouniye B	11	8	72.7	3	3	2	0	0
Total	122	86	70.5	23	12	37	1	13
**Grand total**	**306**	**195**	**63.7**	**43**	**31**	**89**	**1**	**31**

^a^ ITS, Informal Tented Settlements; ^b^ MI, Mixed infections; ^c^ WI, WASH Intervention.

**Table 2:** Distribution and percentages of ST1, ST2 and ST3 isolates among the Lebanese population living in North Lebanon region and the Syrian refugee cohort.

Cohort	*Blastocystis* sp. STs			Reference
	ST1	ST2	ST3	
Lebanon	12 (34.3%)	12 (34.3%)	11 (31.4%)	[35]
Lebanon	35 (25.4%)	39 (28.2%)	64 (46.4%)	[4]
Lebanon	8 (14.8%)	11 (20.4%)	35 (64.8%)	[16]
Lebanon	13 (26.0%)	12 (24.0%)	25 (50.0%)	[36]
Total Lebanon	68 (24.5%)	74 (26.7%)	135 (48.8%)	
Syrian refugees	43 (26.4%)	31 (19.0%)	89 (54.6%)	Present study

## Data Availability

Not applicable.
